# Application of Plackett-Burman Experimental Design for Lipase Production by *Aspergillus niger* Using Shea Butter Cake

**DOI:** 10.5402/2013/718352

**Published:** 2013-02-13

**Authors:** Aliyu Salihu, Muntari Bala, Shuaibu M. Bala

**Affiliations:** ^1^Department of Biochemistry, Ahmadu Bello University, Zaria, Nigeria; ^2^Department of Biochemistry, Bayero University, Kano, Nigeria

## Abstract

Plackett-Burman design was used to efficiently select important medium components affecting the lipase production by *Aspergillus niger* using shea butter cake as the main substrate. Out of the eleven medium components screened, six comprising of sucrose, (NH_4_)_2_SO_4_, Na_2_HPO_4_, MgSO_4_, Tween-80, and olive oil were found to contribute positively to the overall lipase production with a maximum production of 3.35 U/g. Influence of tween-80 on lipase production was investigated, and 1.0% (v/w) of tween-80 resulted in maximum lipase production of 6.10 U/g. Thus, the statistical approach employed in this study allows for rapid identification of important medium parameters affecting the lipase production, and further statistical optimization of medium and process parameters can be explored using response surface methodology.

## 1. Introduction

Lipases (E.C. 3.1.1.3) are enzymes that primarily catalyze the hydrolysis of triacylglycerols and show enormous potentials in catalyzing the synthesis of esters through transesterification, thioesterification, and aminolysis in nonaqueous media [1, 2]. Lipases have been utilized in different industrial processes including detergent formulation, flavor enhancement, treatment of fatty effluents, production of biosurfactants, biopharmaceutical formulations, and biodiesel production [3, 4].

Agroindustrial residues are continuously being generated in vast quantity especially in developing countries and their disposal is associated with several environmental problems [5]. Utilization of these agroresidues and byproducts of agroindustries as nutrient sources for microbial lipase production may reduce the final enzyme production cost, which is one of the major challenges affecting the large-scale production [6, 7]. Some of the agricultural residues reported in the literature for lipase production include brans (wheat, rice, soybean, and barley), oil cakes (soy, olive, gingelly, and babassu), and bagasse (sugarcane) [8, 9].

Lipases can be produced by animals, plants, and microorganisms. However, microbial lipases have been extensively studied due to their interesting characteristics such as stability in organic solvents, action under mild conditions, and high substrate specificity [2, 10].

Fungi are among the best adapted species in the utilization of agricultural residues based on their ability to grow on surfaces of various substrates and penetrate into the interparticle spaces of the solid substrates [11]. Additionally, tolerance to minimal water condition renders fungi to be more efficient in the bioconversion of several renewable substrates [12]. *Aspergillus niger* has been used in the production of microbial enzymes including lipases. Since lipase production is inducer dependent, the requirement of a lipid carbon source is very critical for high enzyme yield [4].

Shea butter cake (SBC) is obtained after the extraction of shea butter from the kernel and has been found to contain some residual phenolic compounds, tocopherols, and saturated and unsaturated fatty acids in greatly varying proportions [13, 14]. These attributes make SBC a suitable substrate for production of different value-added products.

In this context, this study was aimed at selecting the significant medium components using SBC as the main substrate through Plackett-Burman (PB) experimental design for lipase production by *A. niger*. This design was selected based on its ability to screen and evaluate the relevant medium components that affect the lipase production, so as to generate reliable and more manageable set of components, as well as indicating how each component affects the overall response [15, 16]. Although many reports have been published on lipase production using different agricultural residues, to the best of our knowledge the literature contains no reports on the use of SBC for extracellular lipase production.

## 2. Materials and Methods

### 2.1. Sample and Microorganism Collection

Shea butter cake (SBC) was obtained following the extraction of shea butter fat by local producers in Zaria, Nigeria. The cakes were washed, milled, and sun-dried. The dried milled samples were sieved through standard mesh sieves to obtain 1 mm particle sizes and were stored at room temperature. *Aspergillus niger* was obtained from the culture stock of Department of Crop Protection, Ahmadu Bello University, Zaria, Nigeria.

### 2.2. Fungal Strain and Inoculum Preparation

Seven-day-old potato dextrose agar plate containing *A. niger* was used to prepare the inoculum culture. The method reported by Alam et al. [17] was employed, where each culture plate was washed with 25 mL of sterile distilled water using bent glass rod and the suspension was filtered through Whatman No. 1 filter paper to remove the mycelia from spore suspension. The spores were counted using the haemocytometer to maintain the inoculum concentration of 1 × 10^8^ spores per mL.

### 2.3. Fermentation Media Preparation Based on Plackett-Burman Design

Twelve experimental runs were generated using Design-Expert software (version 6.0.8, Stat-Ease Corporation, USA) for 11 media components; these are glucose and sucrose as carbon sources, tween-80 and olive oil as inducer sources, and peptone, yeast extract, (NH_4_)_2_SO_4_, and NaNO_3_ as nitrogen sources, while inorganic mineral sources include Na_2_HPO_4_, MgSO_4_, and CaCl_2_. Each component was tested at two concentration levels, low (−1) and high (+1), and the concentration ranges taken for the components were 0–0.5% for glucose, sucrose, peptone, yeast extract, and tween-80; 0–1.0% for olive oil; 0–0.1% for NaNO_3_, CaCl_2_, and MgSO_4_; and 0–0.2% for (NH_4_)_2_SO_4_ and Na_2_HPO_4_. These concentration levels were decided based on several literature reports on lipase production using different substrates. Thus, the experimental range and levels of the nutritional components used in the screening design (PB) in terms of actual and coded values are represented in Table 1. The experiments were carried out according to the design matrix in 250 mL conical flasks. The initial moisture content, temperature, inoculum concentration and particle size were maintained at 60%, 30°C, 5%, and 1 mm, respectively.

### 2.4. Lipase Production by Solid State Fermentation

Twenty grams of SBC (1 mm particle size) was weighed into a 250 mL Erlenmeyer flask, and the medium components of varying concentrations were added into the flask based on the PB design matrix to achieve a desired moisture level of 60%. The contents were thoroughly mixed and autoclaved at 121°C (15 psi) for 20 min. The medium was inoculated with 5% (v/w) *Aspergillus niger* inoculum and incubated at 30°C for 7 days. After the fermentation, sodium phosphate buffer (0.05 M, pH 7.0) was added to each flask, the mixture was shaken on a rotary shaker (180 rpm) for 1 hour at room temperature and the suspension was centrifuged at 5000 ×g for 10 min. The supernatant obtained was used to assay for the enzyme activity.

### 2.5. Determination of Lipase Activity

Titrimetric method was used for determining the lipase activity as described by Freire et al. [18]. An emulsion (18 mL) of olive oil (10%) and acacia gum arabic (5%) in 0.05 M sodium phosphate buffer at pH 7.0 was incubated with a sample (2 mL) of the enzyme extract at 37°C for 15 min in a temperature-controlled orbital shaker. This was then followed by addition of acetone and ethanol (1 : 1) to stop the reaction and to extract the fatty acids. The fatty acids produced were titrated with 0.05 M NaOH. One unit of lipase activity was defined as the amount of enzyme that produced 1 *μ*mol of fatty acids per minute under the assay conditions. The results are expressed in terms of units per gram of SBC (U/g).

## 3. Results and Discussion

Several researches have been carried out on different solid substrates for extracellular lipase production using different fungal species. This study involves the use of SBC as the solid substrate to identify some of the nutritional components contributing to lipase production by *A. niger*. This is due to the fact that oil cakes are among the agricultural residues commonly used for lipase production, since they contain some residual nutrients that can serve as both carbon and inducer sources, and have been reported to be good substrates for microbial enzyme production [19]. 

Plackett-Burman statistical method was performed to screen out positive factors contributing to the production. This will serve as a guide in developing an effective medium composition for enhanced lipase production using SBC as a substrate. The effect of eleven medium components (glucose, sucrose, peptone, yeast extract, tween-80, olive oil, NaNO_3_, CaCl_2_, MgSO_4_, (NH_4_)_2_SO_4_, and Na_2_HPO_4_) was examined. Based on Table 1 which shows the distribution of factors according to the design matrix and the results obtained in this study, highest and lowest lipase production were found to be 3.35 U/g and 0.20 U/g as observed in runs 11 and 9, respectively. This indicates strong influence of nutritional components on the lipase production using SBC. It is not surprising that run 9 appeared to have the lowest production value because no exogenous addition of any nutritional components has been incorporated into it, suggesting that SBC contains some basal nutrients contributing to the overall lipase production. 

Considering the results obtained for the screening experiment, lipase production at this stage is promising when compared with literature reports by several researchers. Rajendran et al. [20] applied PB statistical experimental design to screen twelve medium components based on sixteen experimental trials in developing the fermentation medium for lipase production by *Candida rugosa. *Glucose, olive oil, peptone and FeCl_3_ · 6H_2_O were found to contribute significantly with maximum lipase production of 3.8 U/mL. Also, medium components for lipase production by *Rhizopus arrhizus* were screened using PB experimental design. The most contributing factors that led to a maximum lipase activity of 3.98 U/mL were found to be olive oil, peptone, KH_2_PO_4_, CaCl_2_ · 2H_2_O, and MgSO_4_ · 7H_2_O [21]. In case of solvent-tolerant *Pseudomonas aeruginosa*, addition of 11 nutritional components (glucose, glycerol, xylose, and gum arabic as carbon sources; peptone, tryptone, NaNO_3_, and NH_4_Cl as nitrogen sources; MgSO_4_, NaCl, and yeast extract as vitamin and mineral sources) which were considered to be important for lipase production was screened using PB design. All the components with the exception of NH_4_Cl, glycerol, NaCl, and xylose contribute to the production, with a maximum lipase activity of 2.48 U/mL [15].

**Figure 1:**
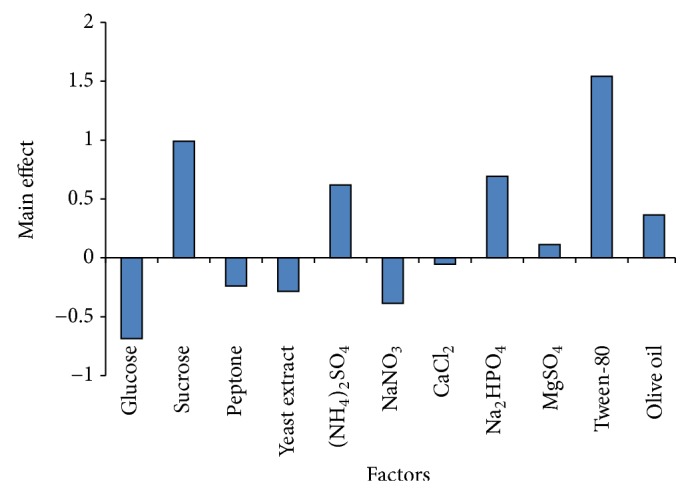
Main effects of the nutritional components on *A. niger *lipase production based on PB experimental results.

The effect of each nutrient component on lipase production was represented in Figure 1. The main effect was estimated based on the difference between the sum of responses obtained at the high level (+1) and at the low level (−1) of each component. It can be seen clearly that the six components (sucrose, (NH_4_)_2_SO_4_, Na_2_HPO_4_, MgSO_4_, tween-80, and olive oil) were found to significantly enhance lipase production while the remaining five components (glucose, peptone, yeast extract, NaNO_3_, and CaCl_2_) have a negative influence on the lipase yield. In contrast to the findings of this study, production of enantioselective lipase by *A. niger* strain AC-54 using wheat bran as a substrate showed that glucose, yeast extract, peptone, NaH_2_PO_4_, KH_2_PO_4_, and water contents are the most significant factors [22]. 

**Figure 2:**
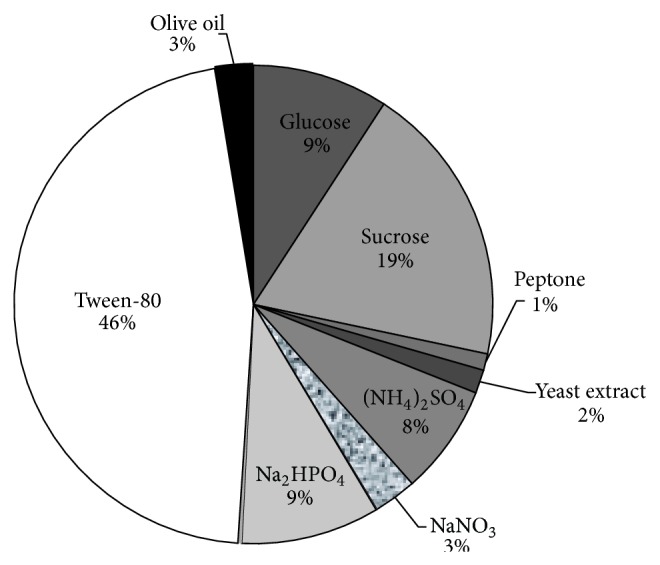
Pie chart representing the percentage contribution of the nutrient components.

Figure 2 shows the percentage contribution of the nutrient components. The results revealed that tween-80 and sucrose are the most contributing components with 46% and 19%, respectively. Thus, tween-80, (nonionic surfactant with a molecular structure polyoxyethylene sorbitan monooleate) has been reported to act as an inducer during lipase production, owing to its potential in increasing cell wall permeability and/or releasing cell bound enzymes [23]. Furthermore, the oleic acid present in its molecular structure gives it the ability to act as a carbon source for extracellular lipases. In the same vein, sucrose, (disaccharide containing glucose and fructose) contributes greatly to lipase production (Figure 2) and showed positive effect (Figure 1). Based on this, sucrose appeared to support the growth and metabolism of *A. niger*, and hence, it is a better carbon source than glucose which instead contributes negatively to the overall lipase activity. This is in agreement with the work of de Azeredo et al. [24] which showed that different carbon sources, mainly carbohydrates and lipids, can support the growth and lipase production by *Penicillium restrictum* using both submerged and solid state fermentation techniques.

Although the PB experimental design employed in this study does not give information on the exact quantity of components to be used in further experiments, it effectively provides general information on each investigated factor. Based on these, the most contributing factor, that is, tween-80 was studied further to determine its optimum concentration.

### 3.1. Effect of Different Concentrations of Tween-80 on Lipase Production

**Figure 3:**
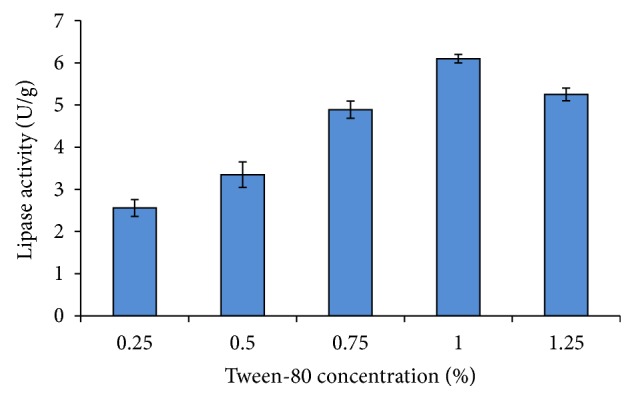
Effects of different concentrations of Tween-80 (0.25–1.25%, v/w) on lipase activity by *A. niger* using SBC.

In order to determine the effect of different concentrations of tween-80 on lipase production using SBC by *A. niger*, the factors contributing positively were maintained at their fixed values, while the negatively contributing ones were omitted from the medium. It can be observed from Figure 3 that lipase activity increases from 3.35 to 6.10 U/g with the increase in tween-80 concentration from 0.5 to 1.0%, but the activity decreased thereafter. This observation is supported by the findings of Maliszewska and Mastalerz [25] in which tween-80 stimulated lipase production by *P. citrinum *but exerted no inhibitory effect (up to a concentration of 0.7%) on lipase activity. Moreover, the presence of tween-80 stimulated *Pseudozyma hubeiensis* HB85A lipase activity by 150.8% [26].

## 4. Conclusion

The findings of this study showed the importance of using PB experimental design as a preliminary optimization technique, which aids in screening and evaluating the medium components affecting the lipase production using SBC by *A. niger*. Among all the tested nutritional components, tween-80 was the most contributing. Based on the results, the substrate (SBC) and *A. niger* which is among the microorganisms generally recognized as safe (GRAS) make this process worthy of future investigation. As such, further statistical optimization using response surface methodology of medium and process parameters needs to be conducted. 

## Figures and Tables

**Table 1:** Plackett-Burman experimental design matrix for screening of various nutritional components for lipase production using SBC as the main substrate.

Run	G % (w/v)	S % (w/v)	P % (w/v)	YE % (w/v)	(NH4)_2_SO_4_ % (w/v)	NaNO_3_ % (w/v)	CaCl_2_ % (w/v)	Na_2_HPO_4_ % (w/v)	MgSO_4_ % (w/v)	Tween-80 % (v/v)	Olive oil % (v/v)	Lipase activity (U/g)
1	0.5 (+1)	0.0 (–1)	0.5 (+1)	0.5 (+1)	0.0 (–1)	0.1 (+1)	0.0 (–1)	0.0 (–1)	0.0 (–1)	0.5 (+1)	1.0 (+1)	0.51
2	0.0 (–1)	0.0 (–1)	0.5 (+1)	0.5 (+1)	0.2 (+1)	0.0 (–1)	0.1 (+1)	0.2 (+1)	0.0 (–1)	0.5 (+1)	0.0 (–1)	2.74
3	0.0 (–1)	0.0 (–1)	0.0 (–1)	0.5 (+1)	0.2 (+1)	0.1 (+1)	0.0 (–1)	0.2 (+1)	0.1 (+1)	0.0 (–1)	1.0 (+1)	1.32
4	0.5 (+1)	0.5 (+1)	0.0 (–1)	0.5 (+1)	0.0 (–1)	0.0 (–1)	0.0 (–1)	0.2 (+1)	0.1 (+1)	0.5 (+1)	0.0 (–1)	2.57
5	0.5 (+1)	0.0 (–1)	0.5 (+1)	0.0 (–1)	0.0 (–1)	0.0 (–1)	0.1 (+1)	0.2 (+1)	0.1 (+1)	0.0 (–1)	1.0 (+1)	0.39
6	0.0 (–1)	0.5 (+1)	0.5 (+1)	0.0 (–1)	0.2 (+1)	0.0 (–1)	0.0 (–1)	0.0 (–1)	0.1 (+1)	0.5 (+1)	1.0 (+1)	3.59
7	0.5 (+1)	0.0 (–1)	0.0 (–1)	0.0 (–1)	0.2 (+1)	0.1 (+1)	0.1 (+1)	0.0 (–1)	0.1 (+1)	0.5 (+1)	0.0 (–1)	1.35
8	0.0 (–1)	0.5 (+1)	0.5 (+1)	0.5 (+1)	0.0 (–1)	0.1 (+1)	0.1 (+1)	0.0 (–1)	0.1 (+1)	0.0 (–1)	0.0 (–1)	0.34
9	0.0 (–1)	0.0 (–1)	0.0 (–1)	0.0 (–1)	0.0 (–1)	0.0 (–1)	0.0 (–1)	0.0 (–1)	0.0 (–1)	0.0 (–1)	0.0 (–1)	0.20
10	0.5 (+1)	0.5 (+1)	0.5 (+1)	0.0 (–1)	0.2 (+1)	0.1 (+1)	0.0 (–1)	0.2 (+1)	0.0 (–1)	0.0 (–1)	0.0 (–1)	1.19
11	0.0 (–1)	0.5 (+1)	0.0 (–1)	0.0 (–1)	0.0 (–1)	0.1 (+1)	0.1 (+1)	0.2 (+1)	0.0 (–1)	0.5 (+1)	1.0 (+1)	3.35
12	0.5 (+1)	0.5 (+1)	0.0v	0.5 (+1)	0.2 (+1)	0.0 (–1)	0.1 (+1)	0.0 (–1)	0.0 (–1)	0.0 (–1)	1.0 (+1)	1.15

G: glucose; S: sucrose; P: peptone; YE: yeast extract; (−1) indicates the low level and (+1) indicates the high level.
